# Methods for analysis of the cancer microenvironment and their potential for disease prediction, monitoring and personalized treatments

**DOI:** 10.1007/s13167-012-0140-3

**Published:** 2012-03-22

**Authors:** Carl-Magnus Clausson, Ida Grundberg, Irene Weibrecht, Mats Nilsson, Ola Söderberg

**Affiliations:** 1 Department of Immunology, Genetics and Pathology, Science for Life Laboratory, Rudbeck Laboratory, University of Uppsala, S-751 85, Uppsala, Sweden

**Keywords:** PLA, Padlock probes, Tumor microenvironment, Personalized medicine, Diagnosis, Prognosis

## Abstract

A tumor does not consist of a homogenous population of cancer cells. Therefore, to understand cancer, the tumor microenvironment and the interplay between the different cell types present in the tumor has to be taken into account, and how this regulates the growth and survival of the cancer cells. To achieve a full picture of this complex interplay, analysis of tumor tissue should ideally be performed with cellular resolution, providing activity status of individual cells in this heterogeneous population of different cell-types. In addition, in situ analysis provides information on the architecture of the tissue wherein the cancer cells thrive, providing information of the identity of neighboring cells that can be used to understand cell-cell communication. Herein we describe how padlock probes and in situ PLA can be used for visualization of nucleic acids and protein activity, respectively, directly in tissue sections, and their potential future role in personalized medicine.

## The cancer cell

Progression from a normal cell to a cancer cell usually requires several genetic alterations including overexpression or alterations of oncogenes and loss of tumor suppressor genes [1]. Whole genome sequencing studies reveal that hundreds of genes are more or less frequently mutated in cancer [2]. Most mutated genes are only altered in a small subset of patients with a particular cancer disease, while only a few genes are commonly mutated. However, collectively rare mutations make up a large proportion of the mutational landscape of cancer. While the DNA sequence acts as a blueprint in the molecular assembly of an individual, the functionally active components of a cell are RNA and proteins. Analysis of RNA and protein are therefore needed to determine to what extent the genetic information is utilized to promote growth and survival of cancer cells. However, the question is whether genetic alterations propagating into expressed RNA and proteins are the sole factor causing and sustaining cancer, and how much this is influenced by epigenetic alterations [3], or if there are additional external factors. To what extent do cells determine their own fates?

## Cancer and microenvironment

For all multicellular organisms intercellular communication is essential to ensure proper organization of individual cells in tissues and organs. The signals transferred between cells provide information that determines the fate of the recipient cells, regulating growth, survival and differentiation. In order to decide to which signals they should respond, cells express different panels of receptors that subsequently will relay the signals, via intricate networks of protein-protein interactions where the activity status of the individual proteins are regulated by post-translational modifications (PTMs). The observed genetic and epigenetic alterations in cancer cells act to promote sustained growth and survival and to overcome the growth restraints from the surrounding cells. The extent of the interplay between cell types, and the regulatory effects from the neighboring cells on the phenotype of a cancer cell, is maybe best exemplified by teratomas. When teratoma cells are introduced into blastocysts of pseudo-pregnant mice they will normalize their phenotype and generate a mosaic that will be tumor free [4,5]. When teratoma cells are instead transplanted into the sides of 129/SV mice, they will form tumors. In fact, transplanting induced pluripotent stem cells (iPSC) into mice to form teratomas is presently used as a step for assessing the validity and viability of iPSC [6-8].

While the normal cells surrounding cancer cells sometimes fail to prevent the cancer from proliferating, this prevention seemingly succeeds more often than it fails as dormant and unnoticed cancer are developed throughout life [9]. This was indicated already in 1934 when small carcinoma was discovered at surprisingly high levels (14%) in prostates of individuals exceeding 50 years of age having died from unrelated causes [10]. The prevalence was discovered to be even higher (34%) in a later study examining the entire prostate glands of even younger males [11]. Apparently the pathogenic transformation of a cell into a carcinogenic state is not enough for full-blown tumor growth. Functions external to the cancer cell must somehow influence its capability to proliferate. Thus, properties of the microenvironment have been gaining interest when trying to understand what activates carcinoma into proliferation. It is now evident that cancerous tumors are not the homogeneous collections of cells they were once thought to be, but consists of several types of cell subpopulations (reviewed by Hanahan and Weinberg [12]). These are cancer cells, cancer stem cells, endothelial cells, pericytes, immune inflammatory cells, cancer-associated fibroblasts, and stem and progenitor cells of the tumor stroma [12]. Together they make up the tumor microenvironment but they have different origins, with the cancer cells acting as tumor initiators. The stem and progenitor cells of the tumor stroma, the immune inflammatory cells and the bone marrow-derived vascular progenitor cells are all recruited from circulation or neighboring tissues. This is true also for the cancer-associated fibro-blasts, which can be recruited myofibroblasts or reprogrammed variants of normal tissue-derived fibroblastic cells. Inflammatory cells can be attracted to the tumor by cancer cells which sometimes undergo necrosis, instead of healthy apoptosis [13,14], creating an inflammatory environment that can both antagonize and promote the tumor growth, and in this light tumors have been described as wounds that never heal [15,16]. Formation of vasculature is regulated by the balance between secreted pro- and anti-angiogenic factors, supporting the cancer cells with nutrients and oxygen. However, the tumor vessels display abnormal organization that impairs transport of nutrients and oxygen, and facilitates migration of cancer cells [17]. The shuttling of cancer cells between the vascular system (i.e. circulating tumor cells (CTC)) and the tumor tissue has been suggested as an important event (i.e. cancer self-seeding) that promotes the growth of a tumor, as the cancer cells are adapted to the local microenvironment [18]. Some of the CTC will fail in the homing to the local microenvironment and will end up in circulation, where they may colonize other sites, supported by interactions with leukocytes that promote extravasation and survival, giving rise to metastasis [19,20]. Immune cells and bone-marrow derived progenitor cells have also been described to promote angiogenesis in tumors [17,21]. Normal stromal fibroblasts are now recognized as being able to induce carcinomas through paracrine signaling with several growth factors [22]. Mesenchymal stem cells can be recruited to tumors and have been observed to differentiate into several cell types in the tumor stroma, and to exert both tumor promoting and suppressing functions in contradicting studies [23]. The concept of cancer stem cells has been widely used during the past few decades to explain different phenotypes and abilities to generate tumors within tumors. An alternative explanation to such events is that the cancer cells experience high plasticity and that differences observed are contingent on the position of the cells in the microenvironment [24,25], to which they adapt to by epigenetic reprogramming, i.e. epithelial-mesenchymal transition (EMT) and mesenchymal-epithelial transition (MET). This is a not yet fully understood program comprising the transition of epithelial cells into cancerous states of varying degrees [12]. Hypoxia or overexpression of hypoxia-inducible factor-1α (HIF-1α) has been shown to promote EMT and a metastatic phenotype [26], suggesting that poor or deficient vascularization of a tumor might induce cancer stem cell properties. The transition into a mesenchymal phenotype is accompanied by epigenetic alterations that activate mesenchymal genes while expressions of epithelial genes are suppressed. To allow repetitive transitions between epithelial and mesenchymal phenotypes bivalent modifications of the promoters by H3K4 methylation and H2K27 trimethylation will keep genes downregulated during EMT, but poised for activation in subsequent MET [27].

It seems that the tumor microenvironment is highly intricate, being made up from numerous cell types all affecting each other in both pathological and normal manners. How may we untangle these interactions among cells and their internal constituents? What methods are needed?

## Requirements on analytical methods

The deregulation of signals in cancer cells, promoting growth and survival to later form tumors, can originate from several levels; the DNA sequence, the epigenetic status, the degree of gene and protein expression, and also protein activity (interactions and post-translational modifications (PTMs)). To obtain a coherent view of a cell, analysis should ideally be performed at several levels in the same cell. In cancer research, xenografts and cell lines are commonly used as cancer models in the place of primary tumor material--a limited resource, also complicated by heterogeneity in composition and variation between samples. However, the usage of cancer models involves artifacts. Concerning xenografts, the mouse stroma may not support the growth of a human tumor. For cell lines, normally grown in 2D mono cultures on plastic support and having almost unlimited supply of nutrients and oxygen, the adaptation (epigenetic alterations) and evolution (selection of novel genomic aberrations) caused by in vitro culturing, can severely influence the phenotype of the cells [28,29]. However, there are areas of research where cell lines, being homogenous populations and unlimited resources, are suitable. One is in detailed studies of cell signaling kinetics. But one needs to be careful drawing conclusions from cell lines as representatives of tumors in vivo, as the activity status of a cancer cell in vivo will be dependent on where in a tumor it resides as this will influence its exposure to signals, nutrients and oxygen. The emerging view of the heterogeneous cancer microenvironment highlights the need for single cell analysis. Studies of the tumor microenvironment thus need to be performed in clinical tissue samples, which closely represent in vivo states. There are then two alternative approaches: either to isolate the cells of interest and perform the analyses in vitro or perform the analyses in situ, keeping the architecture intact. By micro-dissecting populations of cells, standard analytical methods may be used. However, sensitivity demands increase with decreasing amount of obtained material. In situ analysis on the other hand targets the analyte directly in its normal location, omitting the need to collect cells and purify nucleic acids or proteins. For in situ analysis the available methods are few, the most common ones are fluorescence in situ hybridization (FISH) for analyzing nucleic acids and immunohistochemistry (IHC) for protein detection. These methods are mainly based on the use of probes such as oligonucleotides or antibodies and as a consequence thereof, the selectivity becomes limited due to the inherent crossreactivity of the probes that may generate false positive signals. We will continue with an in depth description of a few in situ methods developed in our research groups that overcome this problem by requiring multiple criteria to be fulfilled for a positive identification, providing single-nucleotide discrimination for detection of nucleic acid and the possibility to visualize the active fraction of a protein, by targeting protein interactions or PTMs. Moreover, simultaneous detection of different biomolecules in situ will help in the understanding of the complex function and regulation of cells. An optimal tool would have the ability to visualize different positions in a signaling pathway and measure phenotypic variations, e.g. expression, within to achieve a more complete picture over what is going on inside a single cell.

## Visualization and genotyping of nucleic acids

**Figure 1:**
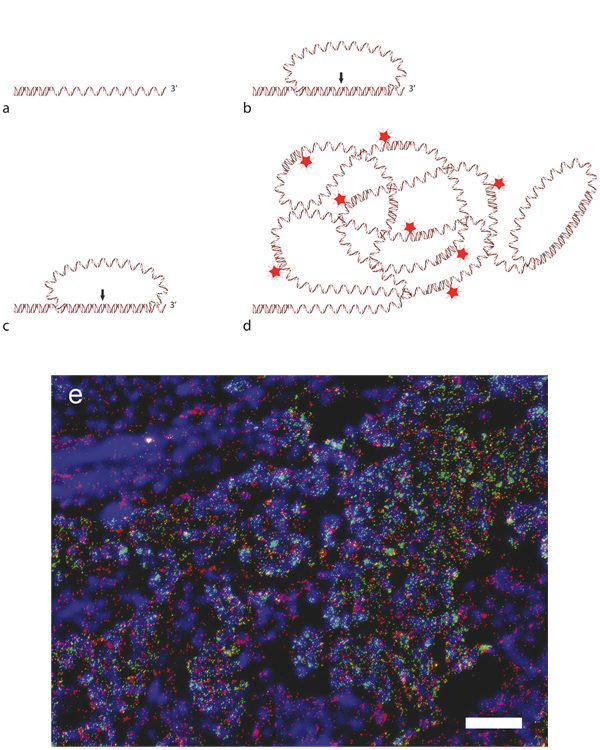
**Detection of nucleic acids with padlock probes**. **a **A single stranded stretch of DNA containing the target site is generated by enzymatic digestion of DNA or reverse transcription of RNA, producing a free 3'-end. **b **A perfect hybridization of a padlock probe will bring the 5'-and 3'- end of the padlock probe together (*arrow*), **c **as the gap is sealed by ligation a circular DNA molecule is created. **d **The free 3'-end of the target sequence will act as a primer for RCA to produce a concatameric RCA-product, complementary to the padlock probe, to which fluorescence-labeled detection oligonucleotides can hybridize, for visualization. **e **Detection of *HER2 *(*green dots*) and *β-actin *(*red dots*) transcripts using padlock probes in fresh frozen tissue sections from breast cancer [38]. Nuclei are counter-stained with DAPI (blue), scale bar equals 50 μm.

Detection of nucleic acids by hybridization of FISH probes [30] has been used for decades but lacks the ability to discriminate between closely similar sequences. To increase the selectivity over what the base pairing of a single probe provides, padlock probes were developed [31]. These are linear oligonucleotides of approximately 70 to 100 nucleotides in length with target-complementary 5'- and 3'- ends which constitute dual target recognition when both probe arms must hybridize correctly to the target. When padlock probes hybridize to their correct target the ends of the padlock probe are brought together in a head to tail orientation, with only a nick in between. The nicks can be sealed by a DNA ligase to create circles that are locked onto the target strands as padlocks [31]. This nick ligation will only occur if there is a perfect match between probe and target at the ligation junction, leaving mismatched probes unligated. Padlock probes were recognized early on for being useful for in situ analysis and the first published application demonstrated genotyping of centromeric sequences [32] using hapten or fluorescence labeled probes. However, with this approach single molecules could not be detected because of high background from unspecifically bound probes. To be able to visualize individual padlock probes, we later used rolling circle amplification (RCA) with the probes as templates. RCA is an isothermal amplification technique of circular DNA molecules that creates long single-stranded DNA molecules with tandem repeats complementary to the original circles [33], here being the padlock probes. The contiguous RCA products will by nature collapse into micrometer-sized DNA-bundles. By targeting the RCA product with fluorophore-labeled oligonucleotides, these can be visualized as bright objects easily distinguishable over background fluorescence [34]. The RCA also selects for circularized padlock probes, as only ligated probes can be amplified, which further enhances the inherent selectivity of the assays. To facilitate the binding of the padlock probe to its target sequence, the DNA needs to be enzymatically prepared in situ, to create a single stranded stretch. This is achieved by combining restriction enzyme digestion with an exonuclease step for target preparation. The free 3'- end of the target strand can then be utilized to prime the RCA and it is thus important that this is located close to the bound padlock probe, creating an RCA product that will be an elongation of the targeted sequence (Figure 1). The method was first described for genotyping of mitochondrial DNA (mtDNA) [35]. By having two padlock probes, one for each genotype (designed with different detection sequences), the wild-type genotype was easily distinguished from the mutant in both homo- and heteroplasmic cell lines as well as in fresh-frozen tissue sections [35].

Genotyping of transcripts and gene expression profiling using padlock probes and RCA has been of great interest. However, direct RNA-templated ligation of DNA padlock probes have been shown to be rather inefficient [36,37]. We circumvented the limitation of poor ligation efficiency on RNA molecules with a conversion of the mRNA into cDNA [38]. In brief, the method is initiated by in situ reverse transcription using LNA-containing primers, followed by digestion of the mRNA part of the mRNA/cDNA duplex for creation of a single-stranded target. Padlock probes are thereafter hybridized to the cDNA transcripts and circularized upon absolute complementarity to the correct targets and finally, RCA is primed from the cDNA to create visible signals, as described above. LNA-modified oligonucleotides have been widely used in several in situ hybridization studies and are known to increase hybridization affinities towards DNA and RNA [39,40]. In addition to substantial improvement of the reverse transcription step, due to increased hybridization efficiency, the LNA-containing cDNA primers also protect the RNA from breakdown by RNase H [41] - a feature herein utilized to anchor the cDNA molecule to the mRNA throughout the whole procedure. Padlock probes will not just yield information about the genotype of the transcript, but will also provide information on target localization in the tissue and cell. The approach has sufficient resolution to detect point-mutations, splice-variations, and fusion transcripts. Another advantage of analysis at the RNA level, which is closer to the phenotype than DNA, is that it provides information about whether the mutation is expressed or over-expressed and whether the wild-type allele is functionally lost (expressed loss-of-heterozygosity). Thus, this RNA-based assay will also capture effects of promoter and enhancer mutations, epigenetic alterations caused by large chromosomal rearrangements and miRNA effects on transcription.

## Visualizing protein interactions and post-translational modifications

The activity status of proteins is in most cases regulated by PTMs, such as phosphorylation, which will cause structural changes in the proteins and thereby expose catalytic sites or promote interactions with other proteins. For localized detection of protein interactions, double-staining of proteins with IHC is commonly used. Employing two binders targeting different proteins and being differently labeled, the co-localized observation of these infers interaction. However, the resolution of a regular fluorescence microscope is 200-350 nm [42], which is arguably far too inferior for a positive identification of interacting molecules in a co-localization assay. One approach to improve the detection of co-localized antibody-coupled fluorophores is to utilize the Förster resonance energy transfer (FRET) that occurs between fluorophores with compatible excitation and emission spectra. The phenomenon requires short distances, below 10 nm, and is dependent on the orientation of the fluoro-phores [43]. For detection of protein interactions in tissue sections the fluorophores needs to be coupled to antibodies targeting the interacting pair of proteins [44,45]. However, the method is currently hampered by low quantum yield, tissue autofluorescence and over bleeding between fluorophores, making detection of low abundant protein interactions challenging.

**Figure 2:**
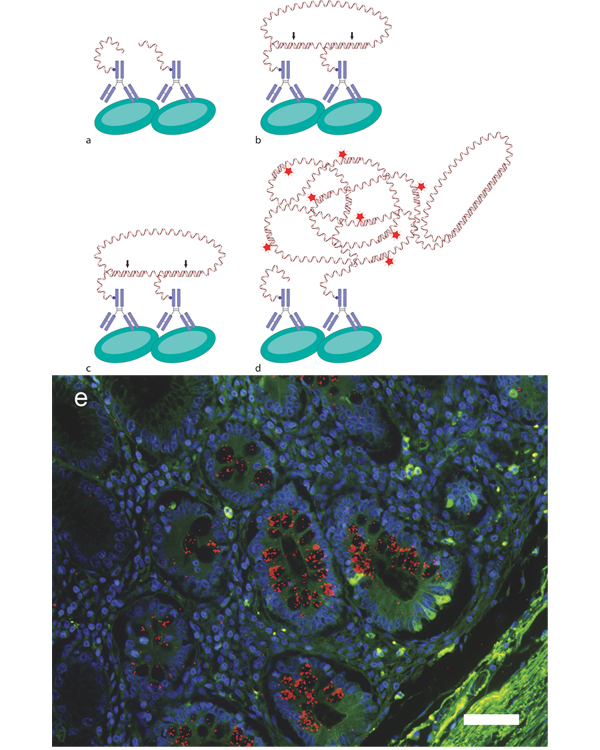
**Detection of protein interactions with in situ PLA**. **a **Proximal binding of PLA-probes to interacting proteins will guide the **b **hybridization of circularization probes, **c **allowing them to be connected by ligation (*arrows*) and thereby creating a circular reporter molecule of the protein interaction. **d **The oligonucleotide on one of the PLA-probes will then act as primer for RCA to generate an RCA product that will be an elongation of the PLA-probe. Fluorescence-labeled detection oligonucleotides are then used to visualize the RCA-product. **e **Detection of Mucin2 glycosylation (Sialyl-Tn) (*red dots*) in formalin-fixed paraffin-embedded tissue sections from intestinal metaplasia [49]. Nuclei are counterstained with Hoechst 33342 (*blue*), using the autofluorescence of the tissue (*green*) to visualize the histology, scale bar equals 50 μm.

To obtain a method that retains the requirement for proximal binding of antibodies to interacting proteins in order to create a signal, as for FRET, but to also provide potent signal amplification, the in situ proximity ligation assay (in situ PLA) was developed [46]. The method utilizes the distance constraints for ligation of two DNA molecules into a reporter molecule and employs PLA-probes which are chimeric molecules consisting of antibodies, for recognizing the protein of interest, equipped with conjugated single stranded oligonucleotides, for subsequent detection purposes. Proximal binding of pairs of PLA-probes to the same target will position the oligonucleotides in close vicinity of each other, which guide the hybridization of two subsequently added circularization oligonucleotides that upon ligation will form a circular DNA molecule. The formation of this DNA circle will thus be strictly dependent on, and will function as a reporter for, the proximal binding of a pair of PLA-probes. In analogy to what is previously described for the padlock probes, the proximity-dependent DNA circle can then be used as a template for RCA, primed by a free 3'-end on one of the PLA-probes, to create an RCA product that will be an elongation of the PLA-probe. The RCA products are visualized with detection oligonucleotides and will be localized close to the protein interaction of interest as they are attached to the PLA probe, which still binds its protein target (Figure 2). The method may be used both to detect protein complexes, by targeting two interacting proteins [46,47], and to determine post-translational modifications of a protein, targeting the core protein and a modified epitope [48]. A big advantage is that the bright signals enable detection in formalin-fixed paraffin-embedded tissue sections [49], which commonly suffers from high autofluorescence. To circumvent autofluorescence entirely, it is possible to replace the fluorophore of the detection oligonucleotide with an enzyme, providing an assay for protein interaction analysis by regular brightfield microscopy [50].

Although the distance requirement to generate an in situ PLA signal is theoretically within ~20 nm (calculated from the size of an antibody and length of the nucleotides), the RCA product produced has a diameter of approximately 1 μm. While the size of the RCA products provides for easy detection, due to the high number of repetitive targets for fluorophore-labeled detection oligonucleotides, it also limits the number of RCA products that can be detected in a cell. A few hundred RCA products per cell are often enough to cause saturation, when discrete objects can no longer be defined. To increase the limited dynamic range it is possible to use a mix of different circularization oligonucleotides, with different sequences for detection oligonucleotides, such that the concentration ratio of the added circularization oligonucleotides will then be reflected among the RCA products. In practice, the effect is that when one type of RCA product labeled with a distinct detection oligonucleotide saturates the area of analysis, it is possible to instead view an RCA product type originating from a circularization oligonucleotide used at a lower concentration, which will be reported by its respective other fluorophore-labeled detection oligonucleotide. By viewing the type of RCA products that are within the optimal dynamic range for each cell, it is possible to simultaneously measure in situ PLA targets in tissue sections containing cells with both high and low levels [51].

## Combined visualization of nucleic acids and proteins

For visualization of interactions between protein and nucleic acid (PNI), to visualize e.g. epigenetic status of individual genomic sequences, things get more complicated as probes targeting both types of biomolecules are needed. To detect a specific DNA-sequence and its co-localization with a particular protein, immunofluorescence (IF) and FISH can be combined to form methods named immuno-DNA FISH and immuno-RNA FISH [52-54]. The protein of interest is targeted with fluorescence-labeled antibodies, while DNA is denatured and the target sequence detected by hybridization of hapten-labeled detection oligonucleotides. These haptens can in turn be detected by IF through utilizing antibodies targeting the hapten label. By superimposing the different fluorescence channels, co-localization of the nucleic acid sequence and the protein can be detected and visualized in situ. Although immuno-DNA/RNA FISH presents a valuable tool for investigation of PNIs in situ, spatial resolution is restricted to the resolution of the microscope used and proteins can only be detected if they are expressed at a certain level or above for IF to distinguish them over background fluorescence. Furthermore, the method cannot distinguish between highly homologous sequences as it is based on hybridization of a detection oligonucleotide. To increase the selectivity, enabling detection of protein-DNA interaction with an SNP-resolution, we developed a method that is based on padlock probes to target the DNA. Upon ligation, the padlock probe is converted into a circularization probe that will be utilized to survey the vicinity for bound PLA-probes. In order to prevent binding of the padlock probe to free PLA-probe, due to sequence complementarity, the padlock probe was equipped with hairpin structures that would hide the complementary sequences during PLA-probe staining. After washes the padlock probes can be activated, by degrading the uracil containing hairpins, thereby enabling hybridization to the bound PLA-probes [55]. After ligation the amplification and detection can be performed as for conventional in situ PLA. With this method we were able to detect proximity between histone H3 and Alu-repeats in human cells. Although the method was demonstrated to be highly selective in discriminating between closely homologous sequences (human and mouse Alu-repeats), it suffers from poor efficiency in targeting genomic DNA. Further efforts to make genomic DNA more accessible, i.e. by alternative fixations and sample pretreatments, are required to enable studies of single copy genes.

## The prospect of personalized medicine

Although both padlock probes and in situ PLA are recently developed methods, we have high hopes that they within a near future will be adopted for routine diagnostics, just as IHC and FISH has become. A big advantage with in situ assays is that they do not require sophisticated and expensive equipment, both assays based on padlock probes and in situ PLA can be used with standard epifluorescence microscopes or, if the RCA-products are developed into chromogenic signals, even brightfield microscopes [50]. In situ analysis with padlock probes can be a rapid test for detection of recurrent mutations that might be useful to guide e.g. therapy choice, and the ability to genotype individual cells in a tumor provides the means to determine clonal evolution. To evaluate the functional consequences different mutations will have on a cell, in situ PLA can be used to determine how these mutations effects the proteins ability to interact with its partners. As mutations often occur at different proteins in a signaling pathway, by monitoring downstream hubs in signaling pathways all mutations with a similar consequence will be detected. In addition, as in situ PLA can be used on both cultured cells and tissue sections, the same assay can be used for drug development [56], diagnosis, selection of therapy and to monitor therapy response.

Considering the complexity of a tumor, the key factor for successful diagnostic assays lies in to what extent they can provide a coherent view of the cellular processes governing the growth and survival of the cancer cells. Methods to determine activity status of proteins, to analyze nucleic acids with a higher resolution and to determine interactions between these types of biomolecules at a single cell level will most likely be pivotal for future molecular pathology as they provide a tool to address cell-cell communication in the complex microenvironment of cancer. By understanding these processes, along with the ability for better characterization of individual patients, new routes for personalized treatment strategies will open up. Although cancer cells become addicted to the growth promoting signals derived from a mutated signaling pathway they still possess the ability to respond to other signals. The heterogeneity within the clone, and contacts with different cells and microenvironment of each individual cancer cells, provide escaperoutes when drugs are used to target one deregulated pathway. Single agent treatment strategies would thus probably have very limited success in eradicating all malignant cells within a patient. Hence, other growth and survival promoting pathways must be analyzed in addition to the ones altered by a mutation. An inherent restriction of single cell analyses is that the possibility to perform repetitive analysis on the same cell is limited. Because of this, multiplexed analyses are requested in order to obtain as much information as possible from each cell, and we have therefore been working on approaches to multiplex in situ PLA (Leuchowius et al., submitted). As padlock probes and in situ PLA utilize the same read-out--visualization of RCA products--it is possible to combine these methods for simultaneous detection of proteins and nucleic acids [57]. This provides a tool for investigation of active signaling both at the levels of signal transduction and expression of target genes.

As discussed previously, the interplay between the different cell types in a tumor will very likely have a major role on the outcome of all individual cancers and methods that identify patients with an adverse tumor cell communication will be important for prediction of disease and selection of therapy. The aberrant vessels formed by tumor angiogenesis may have a larger role in promoting growth of cancers than supporting them with nutrients and oxygen (something that they actually are deficient in), as the loosely assembled vessel walls facilitate intra- and extravasation of cancer cells. This will increase the possibility that CTC home to adjacent sites rather than forming distal metastasis. The hypoxic environment, due to insufficient organization of the tumor vessels, induces EMT that provides the cancer cells with a more motile phenotype. Both padlock probes and in situ PLA will very likely have a substantial role in elucidating the mechanisms involved in the cellular communications in tumors, something that then can be adopted for personalized medicine to predict disease progression, and in development and selection of therapy to restore the tumor microenvironment to a state that does not promote cancer growth. Albeit in situ PLA analysis of epigenetic alterations of individual promoters is not feasible at the moment, improvement in efficiency of the padlock-based in situ PLA [55] might soon provide a tool to investigate epigenetic reprogramming of cancer cells during transitions between epithelial and mesenchymal phenotypes.

Methods for analysis of cancer microenvironments will most likely have a large impact on personalized medicine and, although still early, the methods described herein may provide new opportunities to better predict disease progression and therapy response.

## Conflict of interest

M.N. owns shares in the company Olink Biosciences that commercializes the PLA and padlock probe technologies.
